# Chemistry among Dentists

**Published:** 1858-04

**Authors:** 


*Chemistry among Dentists.*—We believe in Chemistry, of course. It is our own department, and like the apostle, we are disposed to "magnify our office." For that reason we are jealous of its reputation, and do not care to see it committed to the defence of crude theories and mere hypotheses. Whenever we see it so committed, we feel bound at least to enter our protest, lest the cause of science suffer from the silence of her friends, and the truth decline in value from its forced connection with unsound speculations.

These remarks have been called forth by the reports of the proceedings of the Dental Convention of Northern Ohio and the Mississippi Valley Association of Dental Surgeons, at their recent meetings.

We find Dr. Watt quoted as advocating a special line of treatment of infants to prevent dental caries. He proposes to administer phosphate of lime to mothers of children likely to have bad teeth. This is to be done during the latter months of gestation and the earlier months of lactation. Of course, this plan of treatment is based upon the idea that defective teeth are owing to the deficiency of phosphate of lime in the economy, a very gratuitous assumption. If there were any such general deficiency of bone-earth, the bones would manifest it equally with the teeth, and we should have such children presenting the phenomena of rickets—soft and crooked bones, bulging joints, big heads, and all the revolting physiognomy of that distressing malady. Now every one knows that this is not the case. Many a child whose teeth decay, looks stout and hardy, and enjoys, to all appearance, excellent health.

The treatment is an old one for rickets. It has been tried and been abandoned, as utterly inefficient. It was the recommendation of a crude and imperfect chemical theory, which took no account of the countless delicate changes continually going on in the animal organism. There is something more to be considered than the mere question of plus and minus, in animal chemistry. A defective ingredient in any organ cannot be supplied by pouring into the mouth its chemieal equivalent. We must confer upon the suffering organs the vital power in which they are deficient. We must induce them to act upon what is presented to them; otherwise it is as beneficial on the shelves of the apothecary as in the stomach of the patient, and better, perhaps, as then, at least, it can do no harm.

If there is (which remains to be proved) any deficiency of "ossific matter" in those teeth, it arises not from an insufficient supply of phosphates, which is simply an impossibility upon an American diet scale, but from a want of ability on the part of the ultimate elements of the tooth to separate the necessary ingredients from the mass of the blood. It is difficult to imagine how this condition is to be benefitted by the administration of phosphate of lime.

There were a few other odd chemical notions advanced at these meetings, upon which we feel ourselves obliged to make a few passing comments. In speaking of the acids formed in the mouth, and acting upon teeth, we find the glucic quoted as one having a remarkable affinity for lime, and being especially injurious to the teeth. This opinion was advanced in reply to a question as to what acid was generated in consequence of the use of sugar.

This surprised us not a little. The saccharine substances used in food are all made of cane sugar. Glucic acid is formed from glucose or grape sugar, under peculiar circumstances, which do not and cannot exist in the mouth. When glucose is mixed with lime in sufficient quantity, an alkaline liquid results, which, if exposed for some time to atmospheric air, becomes neutral, and no longer lets fall a precipitate upon the addition of carbonic acid. By the action of some acid which has a greater affinity for lime, as the oxalic, the glucic acid is set at liberty. The acid is a rare one, but recently discovered. Its properties are little known. One thing, however, is pretty certain, there is no likelihood of its being brought in contact with the teeth.

As for cane-sugar, it has not been shown that it undergoes any such change in a similar combination. It is susceptible of four kinds of fermentation, the *alcoholic;* the *viscous*, in which a neutral substance is formed; the *lactic*, taking place in the presence of putrefying azotized bodies, and giving rise to lactic acid, a most powerful solvent for phosphate of lime; and the *butyric* in presence of certain ferments altered by the air. The alcoholic fermentation, if prolonged, gives rise to acetic acid. All these changes require time, and if the saccharine fluids were kept about the teeth, there might be some immediate danger to these organs from the free use of sugar; but they are so rapidly removed from the mouth, that we must seek for some other explanation of their injurious action upon the teeth, if indeed they exert any.

Some comments were also made upon the difference between the caries produced by hydrochloric and sulphuric acid, and that caused by nitric acid. Here again we have gratuitons hypotheses, resting upon no basis of observation.

The presence of nitric acid in the mouth has never been demonstrated. The experiments of Dr. Mitchell should not be cited in proof of its presence, to any one who is at all acquainted with the practical difficulties of its detection. It is indeed, occasionally formed under peculiar circumstances, from the oxydation of nitrogenous matters, but the conditions of its formation do not exist in the mouth. It has not been demonstrated to be a constituent of the body; hence the recommendation to limit the quantity of azotized food in cases of white decay is, to say the least of it, rather premature.

Still more remarkable is the recommendation to dispense with salt in cases in which the animal portion of the tooth remains while the calcareous portion is dissolved out, "as hydrochloric acid is the destructive agent in such cases." Now, when we consider that, chemically speaking, no acid can act in any other way than dissolving out the calcareous salts, this appears to be a very unintelligible distinction. The decomposition of common salt in the living body is yet to be demonstrated, and the presence of free hydrochloric acid any where in the animal economy is still an open question.

Equally unfounded is the assumption that lactic acid is the cause of "chemical abrasion," of the teeth. Has any one analyzed the fluids of the mouth in these cases? If so, we have never heard of it, and we believe we are tolerably well acquainted with what is going on in the way of Dental Science. It must be borne in mind too, that no sciolist can grapple with this question. It requires analytical experience of the highest order to attempt the resolution of such problems. No less a chemist than Lehmann confesses that "to determine the presence of lactic acid, is one of the most difficult tasks in analytical animal chemistry."

It is with reluctance we have made these brief comments upon the doctrines advanced in the late meetings of the Western associations, but we cannot allow them to pass without protest. We cannot suffer them to go abroad as representing the opinions of the dental profession in America, full as they are of great chemical errors, and still greater assumptions.

